# Availability of malaria diagnostic tests, anti-malarial drugs, and the correctness of treatment: a systematic review and meta-analysis

**DOI:** 10.1186/s12936-023-04555-w

**Published:** 2023-04-18

**Authors:** Hosein Azizi, Elham Davtalab Esmaeili, Fariba Abbasi

**Affiliations:** 1 Research Centre for Evidence-Based Medicine, Tabriz University of Medical Sciences, Tabriz, Iran; 2 Women’s Reproductive Health Research Center, Tabriz University of Medical Sciences, Tabriz, Iran; 3 Department of Epidemiology and Biostatistics, School of Public Health, Tehran University of Medical Sciences, Tehran, Iran; 4 Road Traffic Injury Research Center, Tabriz University of Medical Sciences, Tabriz, Iran; 5 Department of Diseases Control and Prevention, Vice-chancellor for Health, Tabriz University of Medical Sciences, Tabriz, Iran

## Abstract

**Background:**

Health facilities’ availability of malaria diagnostic tests and anti-malarial drugs (AMDs), and the correctness of treatment are critical for the appropriate case management, and malaria surveillance programs. It is also reliable evidence for malaria elimination certification in low-transmission settings. This meta-analysis aimed to estimate summary proportions for the availability of malaria diagnostic tests, AMDs, and the correctness of treatment.

**Methods:**

The Web of Science, Scopus, Medline, Embase, and Malaria Journal were systematically searched up to 30th January 2023. The study searched any records reporting the availability of diagnostic tests and AMDs and the correctness of malaria treatment. Eligibility and risk of bias assessment of studies were conducted independently in a blinded way by two reviewers. For the pooling of studies, meta-analysis using random effects model were carried out to estimate summary proportions of the availability of diagnostic tests, AMDs, and correctness of malaria treatment.

**Results:**

A total of 18 studies, incorporating 7,429 health facilities, 9,745 health workers, 41,856 febrile patients, and 15,398 malaria patients, and no study in low malaria transmission areas, were identified. The pooled proportion of the availability of malaria diagnostic tests, and the first-line AMDs in health facilities was 76% (95% CI 67–84); and 83% (95% CI 79–87), respectively. A pooled meta-analysis using random effects indicates the overall proportion of the correctness of malaria treatment 62% (95% CI 54–69). The appropriate malaria treatment was improved over time from 2009 to 2023. In the sub-group analysis, the correctness of treatment proportion was 53% (95% CI 50–63) for non-physicians health workers and 69% (95% CI 55–84) for physicians.

**Conclusion:**

Findings of this review indicated that the correctness of malaria treatment and the availability of AMDs and diagnostic tests need improving to progress the malaria elimination stage.

## Background

Worldwide malaria case incidence has decreased by 27% from 2000 to 2015, and from 2015 to 2019 it reduced by less than 2%. Since 2015, it shows a slowing rate of decrease [1]. Recent noteworthy developments have been performed towards malaria elimination worldwide. However, malaria remains a major public health concern in several tropical areas, particularly in countries with a weak health system [2, 3].

The World Health Organization (WHO) highlighted the appropriate and prompt treatment of malaria cases with first-line anti-malarial drugs (AMDs) in the first 24 h after diagnosis, and malaria guidelines include the indicator “percentage of patients with suspected malaria who received a parasitological test” [4]. It needs the availability of malaria diagnostic tests and AMDs in health facilities to prevent fatal outcomes and prevention of re-establishment of malaria in low transmission areas [5]. However, malaria case management remains a significant shortcoming in various settings [6, 7].

Meta-analysis and empirical studies indicated that decreasing healthcare systems readiness and health service providers’ practice in the appropriate malaria case management, unavailability of Rapid diagnostic test (RDT), AMDs, and shortage in early case detection with appropriate malaria diagnostic tests are major concerns to obtain malaria elimination certification and prevention of re-introduction, especially in countries where malaria transmission is low or interrupted [1, 7, 8].

Furthermore, it seems that COVID-19 pandemic imposed great challenges for malaria elimination and surveillance programmes due to the disruption in early case detection and appropriate case management of febrile and suspected malaria cases both in high and low transmission areas and potential re-introduction regions [9–11].

Therefore, evaluating health facilities’ availability for AMDs and diagnostic tests and healthcare providers’ practice in the appropriate malaria treatment is critical for early case detection, malaria surveillance systems, and elimination programmes [12, 13]. This meta-analysis aimed to estimate summary proportions for the availability of malaria diagnostic tests and AMDs, and the correctness of malaria treatment.

## Methods

### Search strategy

The Web of Science, Scopus, Medline, Embase, and Malaria Journal were systematically searched up to 30th January 2023. Grey literature was searched from Open grey, WHO and CDC reports, congress papers, and records. The study searched any records reporting the availability of diagnostic tests and AMDs, and the correctness of treatment of malaria using text words, synonyms, and medical subject headings (MeSH terms).

The initial search included malaria and/or fever or febrile. Then the study search used the relevant MeSH terms and text words related to malaria and febrile diseases in conjunction with(((((((((((((((malaria{Title/Abstract}) OR (fever{Title/Abstract})) OR (febrile{Title/Abstract})) AND (treatment{Title/Abstract})) ) OR (case management)) OR (test)) OR (diagnosis)) OR (rapid diagnostic)) OR (antimalarial)) OR (anti-malarial)) AND (health worker)) OR (healthcare)) OR (provider)) OR (performance)) OR (practice) AND ((exclude preprints{Filter} AND (humans{Filter}) AND (English{Filter})). The study used a relevant search strategy based on each database search option. The reference lists of the retrieved studies, related reviews and international reports, such as WHO were also searched.

### Eligibility criteria

The inclusion criteria were any cross-sectional or descriptive records/articles reporting *the availability of malaria diagnostic tests* (RDT test and/or microscopic) and/or *AMDs* in the health facilities for malaria cases and/or febrile patients, and also assessed health workers’ correctness of treatment at all age groups. The *correctness of malaria treatment* was defined as prescribing the appropriate and recommended dosage of the first-line AMDs for malaria parasite-positive cases; not only prescribing any anti-malarial drugs; particularly artemisinin-based combination therapy (ACT) [14]. Exclusion criteria were editorials, letters, reviews, conference abstracts, and commentaries. We also excluded Knowledge, attitude, and practices (KAP) and qualitative studies, and studies carried out for active case finding and/or screening and assessed the effects of any specific intervention on malaria case management.

### Outcomes

The primary outcome was the availability of malaria diagnostic tests (RDT test and/or microscopic) and AMDs; and the second outcome was health workers’ correctness of treatment with the first-line AMDs for malaria parasite-positive cases.

### Data selection and extraction

Two reviewers (HA, EDE) assessed the eligibility of records independently in a blinded method. The title and abstract were screened at first, and the two reviewers screened and selected relevant full-text articles. Data were extracted based on the pre-specified criteria into an Excel sheet and then transferred to statistical analysis software. The extracted data was the year, authors, country, study design, sample size, number of health facilities in each study, malaria patients, febrile patients, and health worker type and number.

### Quality and risk of bias assessment

The quality and the risk of bias were evaluated using Newcastle-Ottawa Scale [15]. This instrument considered the following parameters including adequate sample size, sampling method and plan (using unbiased and random sampling methods), using appropriate data collection methods, sample representativeness, inclusion/exclusion criteria, adequacy of response rate, and correct and appropriate statistical analysis.

The final scoring system included 11 criteria for rating different risk of bias elements for each eligible article out of 12 scores. Scale weights (unbiased sampling and data collection method had the highest weights) were recommended by authors for each parameter of the scoring system, as proposed in other meta-analyses. Table 1 categorized the studies into three levels of risk of bias including low risk (9–12 points), moderate risk (5–8 points), and high risk (< 5 points) of risk of bias evolution.

**Table 1:** Characteristics of studies included

First author	Year	Country(s)	Study design	Health facility (n)	Health worker type	Health workers (n)	Febrile patients	Malaria patients (n)	Risk of bias
Signorell [33]	2023	Congo	Cross-sectional	144	NR	NR	4208	3702	Low risk
Mohamoud [34]	2022	Somalia	Cross-sectional	106	95% non-physician	150	NR	50	Low risk
Otambo [35]	2022	Kenya	Cross-sectional	30	Physicians and health workers	NR	1131	257	Low risk
Kibira [36]	2021	Uganda	Cross-sectional	30	NR	NR	NR	330	Moderate risk
Abiodun [17]	2020	Nigeria	Cross-sectional	22	Clinicians and nurses	154	1807	431	Low risk
Cohen [20]	2020	sub-Saharan	Cross-sectional	6453	Physicians, paramedical, CHW, and nurses	7268	24,756	7340	Low risk
Garg [37]	2020	India	Cross-sectional	NR	CHWs	241	3087	825	Moderate risk
Aguemon [38]	2018	Benin	Cross-sectional	27	CHWs	93	NR	313	Low risk
Zurovac [21]	2018	Kenya	Cross-sectional	47	Physicians, nurse	182	1224	366	Low risk
Gallay [39]	2018	Tanzania	Cross-sectional	21	Non-physician, other health workers	187	248	140	Low risk
Plucinski [18]	2017	Angola	Cross-sectional	89	CHWs	212	790	293	Low risk
Namuyinga [19]	2017	Malawi	Cross-sectional	105	Medical assistant, nurse, attendant	150	1427	530	Moderate risk
Pulford [40]	2016	Papua New Guinea	Cross-sectional	NR	CHWs (65%) nurse (30%) others	265	771	122	Low risk
Bamiselu [41]	2016	Nigeria	Cross-sectional	144	70% non-physician	432	NR	NR	Low risk
Zurovac [42]	2015	Vanuatu	Cross-sectional	41	Nurse (80%), nurse aids and midwives (20%)	67	226	NR	Moderate risk
Landman [43]	2015	Haiti	Cross-sectional	30	NR	115	257	11	Low risk
Steinhardt [44]	2014	Malawi	Cross-sectional	107	Medical assistant (75%), clinical officer (25%)	136	1747	629	Moderate risk
Rowe [45]	2009	Angola	Cross-sectional	33	Nurses, physicians	93	177	59	Low risk
Total	–	–	–	7429	–	9745	41,856	15,398	–

*NR* not reported; *CHWs* community health workers

### Statistical analysis

STATA version 14.0 (Stata Corp, College Station, TX, USA) was carried out for data analysis. The summary proportions with a 95% Confidence interval (CI) were calculated for the availability of malaria diagnostic tests, anti-malarial drugs, and the correctness of malaria treatment. Pooled proportions of the availability of malaria diagnostic tests, AMDs, and the correctness of treatment were calculated using the Der Simonian and Laird method via the random effects model^15^. Cochran’s Q test and I^2^ were performed for heterogeneity between studies assessing. Sub-group meta-analysis by health worker type was used for the summary proportion of the correctness of malaria treatment. Trend of the correctness of malaria treatment was estimated by considering standard error in each study over the years [1, 16].

## Results

### Study selection and characteristics

**Fig. 1:**
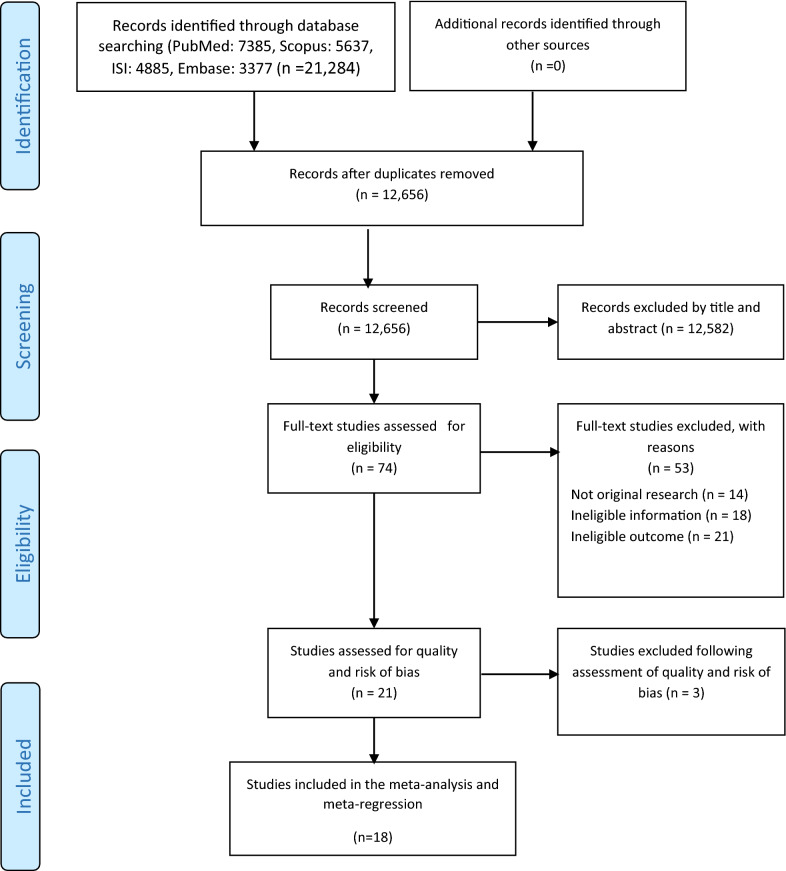
Search results and study selection and inclusion process

A total of 21,284 records were retrieved in the review. Of those 10, 372 records were removed due to duplication. Of which, 12,582 were excluded due to irrelevant titles, abstracts, and texts. In this step, 74 articles were considered for the full-text review. Of which, 53 articles were removed due to not original research, ineligible information, and ineligible outcome. Of which, 3 original studies were excluded due to the high risk of bias assessment. Finally, 18 articles were involved in the meta-analysis (Fig. 1). Of 18 articles, the correctness of malaria treatment was reported in 16 articles, the availability of anti-malarial drugs in 12 studies, and the availability of malaria diagnostic tests (RDT or/or microscopic) in 10 studies.

Table 1 shows that the characteristics of the studies included. All studies included were cross-sectional designs and published between 2009 and 2023 and the majority of studies had been conducted in Africa. It is notable that there were no eligible studies found from low transmission areas or countries in the elimination phase. Although some of the studies included were not reported absolute numbers of the study characteristics, however, a total of 7,429 health facilities (HFs), 9,745 health workers, 41, 856 febrile patients, and 15,398 confirmed malaria patients have participated in the study (Table 1).

### Meta-analysis

**Fig. 2:**
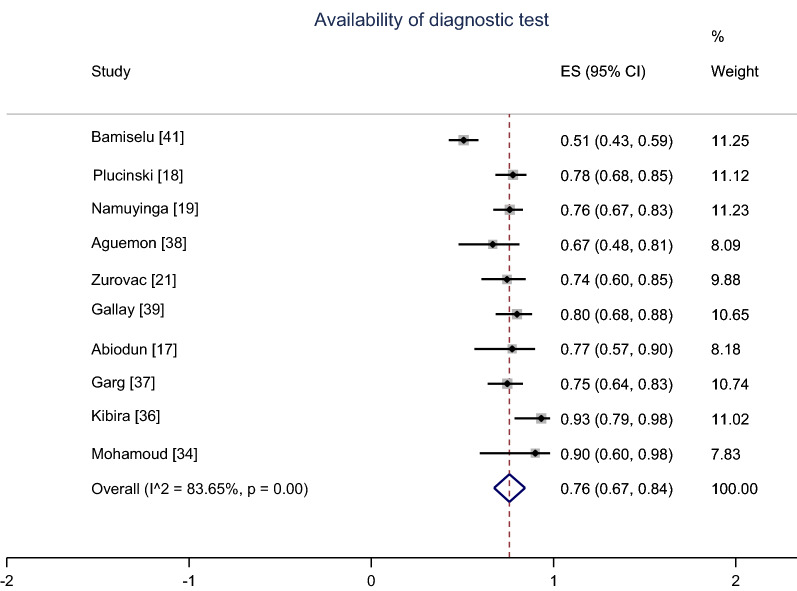
Meta-analysis proportion of the availability of malaria diagnostic tests

**Fig. 3:**
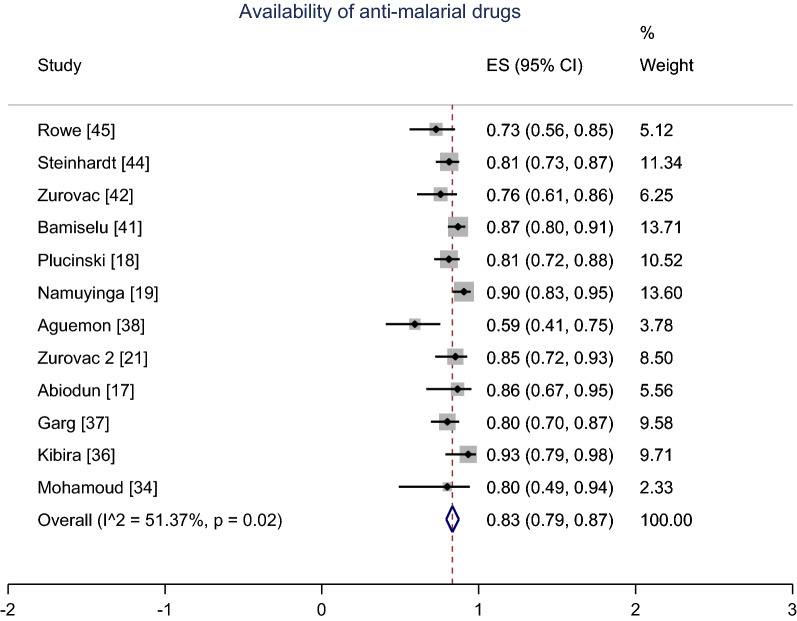
Meta-analysis proportion of the availability of anti-malarial drugs

For the pooling of studies, a meta-analysis using random effects model for 10 studies indicated the summary proportion of the availability of malaria diagnostic tests (RDTs and/or microscopic) in health facilities, 76% (95% CI 67–84%); I^2^ = 83.6% (Fig. 2). Likewise, the pooled proportion of the availability of the first-line AMDs in health facilities using random effects for 12 studies was 83% (95% CI 79–87%); I^2^ = 51% (Fig. 3).

**Fig. 4:**
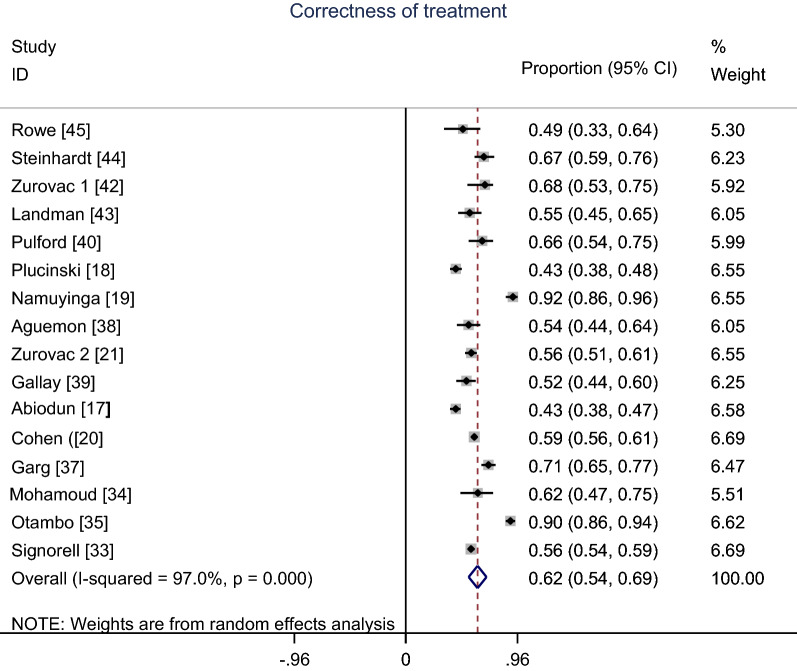
Meta-analysis proportion of the correctness of malaria treatment

Figure 4 shows the meta-analysis proportion of the correctness of malaria treatment with the first-line AMDs. The correctness of malaria treatment varied from 43% in Abiodun et al. [17] and Plucinski et al. [18] studies to 92% in Namuyinga et al. [19] study. A pooled meta-analysis of 16 studies using random effects indicates overall summary correctness of malaria treatment proportion 62% (95% CI 54–69%); I^2^ = 97%.

**Fig. 5:**
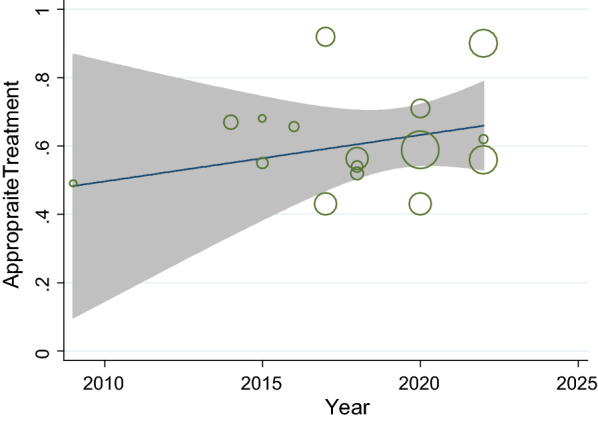
Trend of the proportion of correctness of malaria treatment from 2009 to 2023

**Fig. 6:**
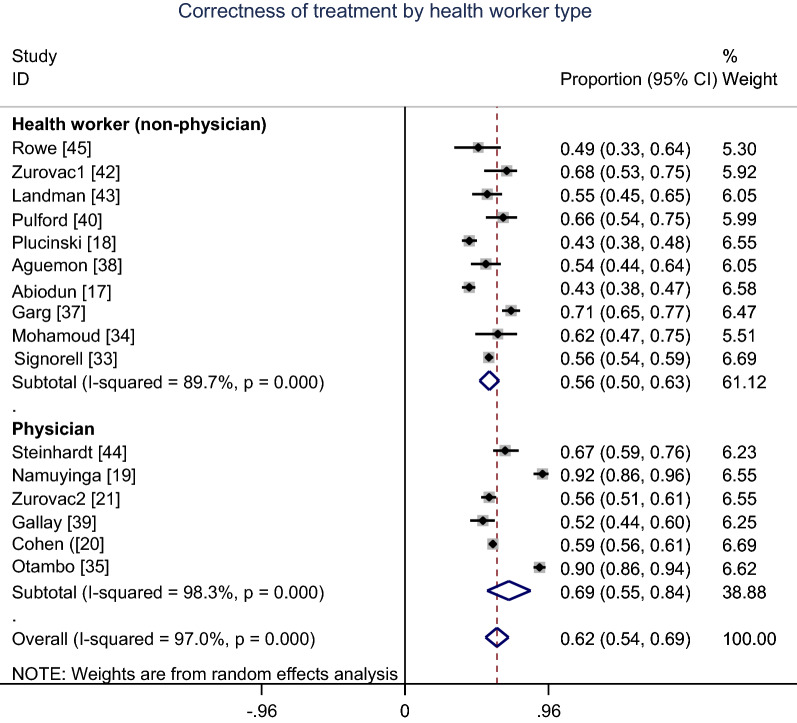
Sub-group meta-analysis proportion of the correctness of malaria treatment by healthcare providers

Overall, the appropriate malaria treatment was improved over time from 2009 to 2023 (Fig. 5). Concerning subgroup meta-analysis proportion of correctness of malaria treatment by health worker type, the pooled meta-analysis using random effects was 53% (95% CI 50–63%; 10 studies) for *non-physicians* healthcare providers and 69% (95% CI 55–84%; 6 studies) for *physicians* (Fig. 6).

## Discussion

Given that malaria testing, the appropriate treatment of confirmed malaria cases with the first-line AMDs, and the availability of malaria diagnostic tests for early case detection are the major components of appropriate malaria case management [8, 14]; this systematic review and meta-analysis was aimed to estimate the pooled proportion of appropriate malaria treatment, availability of AMDs and malaria diagnostic tests in health facilities. The current study findings revealed that no study was conducted in low malaria transmission areas and countries in the elimination phase and all studies included have been conducted in malaria transmission settings.

However, evidence indicated that in low-transmission countries the health system vigilance and health workers’ readiness including awareness and practice in the correct management of suspected malaria was decreased due to the long absence of malaria cases [1]. The prevention of the re-introduction of malaria and malaria elimination programmes are susceptible to serious challenges [7]. Previous findings indicated that to achieve malaria elimination criteria, the malaria surveillance system should be able to detect, manage and report any new malaria cases to the health system; and the malaria surveillance system should be included effective vigilance, which in combination with other components could prevent the re-introduction of malaria transmission [8].

This review found the overall proportion of the correctness of malaria treatment, the availability of AMDs, and malaria diagnostic tests was 62%, 83%, and 76%, respectively. In accordance with the current study, the appropriate malaria treatment was 60% in a study by Azizi et al. [1]. 59% in a study by Cohen et al. [20], and 56% in a study by Zurovac et al. [21]. In a systematic review and meta-analysis, health workers’ compliance with RDT results was 83%, and work experience, patient expectations, health worker type, and perceived effectiveness of the test were related factors [22]. In a meta-analysis study, Kattenberg et al. recommended that RDTs and polymerase chain reaction (PCR) had good performance characteristics to serve as alternatives for the diagnosis of malaria in pregnancy [23]. In a systematic review conducted by Visser et al., the RDT uptake varied widely from 8 to 100%, and the provision of ACT for patients testing positive varied from 30 to 99% [24]. A review study in sub-Saharan Africa found malaria RDTs are generally used well, though compliance with test results is variable [25].

Therefore, this review suggests evaluating health system vigilance and healthcare providers’ readiness in the correct management of suspected malaria in low transmission settings in addition to high transmission settings, and it is a significant component to obtaining malaria elimination certification criteria.

The correctness of malaria treatment with the first-line AMDs in the particular ACT is a major component of the appropriate malaria case management for malaria surveillance systems [26]. Treatment of malaria patients with first-line AMDs and ACT is very important to prevent the development of severe and fatal outcomes [27]. Evidence showed that the case fatality rate of untreated severe malaria has been estimated 13–21% [28]. The WHO places special emphasis on treating all malaria cases with first-line AMDs in the first 24 h after diagnosis [13]. Therefore, the correctness and appropriate malaria treatment depend on the availability of AMDs and diagnostic tests in health facilities and also it needs healthcare providers’ practice in case management and health systems vigilance [29, 30]. Although, early case detection and malaria cases treatment with ACT have been suggested by the WHO in 2006 [31], stocking and availability of ACT and malaria diagnostic tests were incomplete in some included studies.

In the poor availability of RDT, the introduction of the quality assurance system for malaria microscopy, prioritization of microscopy for febrile inpatient management, and increased health facilities availability of malaria RDTs focusing on outpatient malaria screening should be the programmatic and organizational priorities targeting improved diagnostic services in the various settings [17].

This review provided reliable evidence for appraising health system vigilance and healthcare provider practice as also the weakness and strengths of malaria surveillance programmes. Malaria testing and early case detection from suspected febrile cases can increase timely treatment and prevent lethal outcomes, especially in high transmission settings [2, 32]. In low malaria transmission settings, it could timely early case detection of imported cases and provide reliable evidence to measure the prevention of re-establishment of malaria transmission and also WHO elimination criteria [7, 8].

## Limitations

This systematic review and meta-analysis demonstrate the pooled proportion estimate of the availability of malaria diagnostic tests and AMDs in health facilities, and the correctness of malaria treatment by health workers. However, the present study had limitations. The main concern was between-studies heterogeneity due to including studies (with cross-sectional design) from different countries with different malaria surveillance systems and transmission levels may lead to information and reporting bias for estimating the pooled prevalence estimates. However, no study was found from countries with low transmission and/or clear areas in the elimination phase, and all the studies included were conducted in low malaria transmission settings (homogeneous). Moreover, the study used/involved health worker type, and the appropriateness of the study methods, sampling, and conducting (risk of bias) in the sub-group meta-analysis.

## Conclusion

Findings of this review indicated that the correctness of malaria treatment and the availability of anti-malarial drugs and diagnostic tests need improving to progress the malaria elimination stage.

## Recommendations

Establishments of an effective supply chain for malaria diagnostic tests and AMDs, quality-assured diagnostics, ongoing support for healthcare providers to deliver care conferring to the guidelines, and close monitoring of health systems readiness and practices will ultimately determine the attainment of the policy translation continue the importance of practice and quality of appropriate malaria case-management are required.

In low transmission areas and countries in the elimination phase, investigations and in-service training programs are needed to evaluate health systems and healthcare providers’ readiness and practice in the appropriate case management of suspected malaria and prevention of malaria re-establishment.

## Data Availability

The datasets generated and/or analysed during the current study are available from the corresponding author on reasonable request.

## Abbreviations

ACT: Artemisinin-based combination therapy
AMDs: Anti-malarial drugs
CI: Confidence interval
CHWs: Community health workers
HFs: Health facilities
HWs: Health workers
RDTs: Rapid diagnostic tests
WHO: World Health Organization
